# The association between serum estradiol levels and sperm DNA integrity

**DOI:** 10.1002/em.22500

**Published:** 2022-08-25

**Authors:** Viktor Lu, Oscar Svensjö, Jonatan Axelsson

**Affiliations:** ^1^ Reproductive Medicine Centre Skåne University Hospital Malmö Sweden; ^2^ Reproductive Medicine, Department of Translational Medicine Lund University Malmö Sweden; ^3^ EPI@LUND, Department of Laboratory Medicine Lund University Lund Sweden; ^4^ Department of Biology University of Ottawa Ottawa Canada

**Keywords:** body mass index, estrogen, sperm DNA damage, sperm DNA fragmentation, sperm DNA integrity

## Abstract

In men from the general population, BMI has been associated with a lower sperm DNA fragmentation index (DFI). We wondered whether this could be due to estradiol, which is associated with BMI and reported important for sperm function. Our objective was to investigate the association between estradiol and DFI. In 2008–2010, we recruited 284 young men from the general population to deliver samples of semen and blood and answer questionnaires. Serum concentrations of reproductive hormones and DFI were analyzed, the latter using the Sperm Chromatin Structure Assay. Associations were studied using general linear models. The first model utilized metric values of estradiol, whereas the second model compared men with high and low levels, dichotomized by the median value. A possible interaction between estradiol and testosterone was also examined. When investigating metric estradiol levels and DFI, an inverse association was seen without adjustments (*p* = .02), but the statistical significance was lost at adjustments for potential confounders (*p* = .08). Men with lower estradiol levels (<88 pmol/L, mean 71 pmol/L) had a statistically significantly higher DFI than men with higher levels of estradiol (≥88 pmol/L, mean 110 pmol/L). Mean ratio difference was 1.21 (*p* = .002) without adjustments and 1.18 (*p* = .01) with adjustments. A statistically significant difference in DFI was observed in men with testosterone levels below median when comparing high and low estradiol (*p* < .001). This study supports the idea that serum estradiol levels are protective for sperm DNA integrity, at least at lower testosterone levels.

## 1 INTRODUCTION

Fragmentation of sperm DNA is associated with a lower rate of conception (Evenson & Wixon 2006, Agarwal et al., 2022) and may be a risk factor for adverse health effects in the offspring, such as via mutations (Aitken, 2017; Qiu et al., 2020; Swayne et al., 2012; Wyrobek et al., 2006). One risk factor for sperm DNA fragmentation was reported to be obesity, according to two review articles (Campbell et al., 2015; Liu & Ding, 2017), and bariatric surgery in obese men has been reported to be associated with reductions in sperm DNA damage (Wood et al., 2020). However, a more recent review than the previously mentioned, focusing on specifically BMI and sperm DNA fragmentation found insufficient data of an association between BMI and sperm DNA fragmentation (Sepidarkish et al., 2020). Moreover, overweight men from the general population (unlike many studies performed in subfertile men), were reported to have a reduced risk of fragmentation of sperm DNA (Bandel et al., 2015). Since an elevated BMI may be associated with higher estradiol levels (Kahn & Brannigan, 2017; MacDonald et al., 2010; Vermeulen et al., 2002), and since estrogens are reported to play an essential role in the production and maturation of spermatozoa (Carreau & Hess, 2010; Dostalova et al., 2017) as well as to protect testicular cells against oxidative damage (Hamden et al., 2008), we speculated that lower sperm DNA fragmentation in the overweight men (Bandel et al., 2015) could be due to higher levels of estradiol. This would be in line with a reduction in sperm DNA fragmentation reported in mice treated with low levels of estradiol (Mohammadzadeh et al., 2021), possibly due to either the antioxidant effect mentioned (Hamden et al., 2008) or due to positive effects on the sperm DNA condensation and packaging (Cacciola et al., 2013). Such a mechanism would corroborate the emerging role of estrogens in the function of germ cells (Correia et al., 2015) and also associations reported between levels of estradiol and decreased sperm DNA fragmentation in two previous human studies (Meeker et al., 2008; Richthoff et al., 2002). As indicators of the role that estrogens may play for male germ cells, estrogen receptors (ERs) are present on all cells involved in the process of sperm production (Schulster et al., 2016)

Nonetheless, it may be argued that an association between estradiol and low‐sperm DNA fragmentation could in fact be due to an association between testosterone and lower levels of sperm DNA fragmentation, since higher levels of testosterone may be associated with higher levels of estradiol (Finkelstein et al., 2013)

Accordingly, we wanted to investigate whether estrogen levels in young Swedish men were associated with the sperm DNA fragmentation index (DFI) according to the Sperm Chromatin Structure Assay (SCSA) (Evenson et al., 2002), taking levels of testosterone into account. This was done to possibly explain the association between overweight and lower sperm DNA fragmentation in part of the men from the general population previously studied (Bandel et al., 2015)

## 2 MATERIALS AND METHODS

### 2.1 Study population

During 2008–2010, 25% of all Swedish 18‐year‐old males were recruited to undergo a medical examination at the National Service Administration in Sweden (NSAS) for a potential military service. All the 1681 men who underwent a medical examination at NSAS and lived within <60 km of Malmö were asked to participate in the study. Additional inclusion criteria were to be born and raised in Sweden and to be raised by mothers born and raised in Sweden. Of the 1681 men asked, 241 men (14%) chose to participate in the study. Data was collected from the December 1, 2008 to the May 27, 2010. In addition to these 241 men, another 73 men aged 17–20 years, who fulfilled the above mentioned inclusion criteria regarding background and residence, were recruited through friends of participants and advertisements at schools, giving a total of 314 participants (Axelsson et al., 2011). Due to some missing information regarding abstinence time, DFI, alcohol consumption and medical treatment, and difficulty classifying smoking habits, 30 men were excluded from the analysis. The remaining 284 participants were included.

### 2.2 Venous blood samples

Venous serum blood samples from the participants were drawn before noon. The analysis of hormone levels was conducted at the clinical chemistry laboratory of Skåne University hospital, Sweden. Two methods of analysis were used: (Delfia Perkin‐Elmer) an immunofluorometric method, to measure serum concentrations of estradiol, and ElectroChemiLuminiscenceImmunoassay (Roche Cobas) to measure testosterone, luteinizing hormone (LH), follicle‐stimulating hormone (FSH) and SHBG levels. The coefficient of variance and level of detection for each hormone is previously described in detail (Axelsson et al., 2015). The levels of free testosterone were calculated from serum concentrations of SHBG and testosterone according to Vermeulen et al (Vermeulen et al., 1999).

### 2.3 Physical examination and questionnaire

All subjects were asked to fill in a questionnaire regarding factors that might affect their reproductive function such as smoking habits, alcohol consumption (number of medium‐strong or strong beer; glasses of wine; or glasses with 4 cl strong spirit according to a Swedish standard [Systembolaget, 2022]) and medical treatment the last three months. Participants also underwent physical examination. Weight and height were examined, and BMI was calculated as weight divided by height squared (Axelsson et al., 2011).

Sixty men (21%) reported that they smoked cigarettes, and 47 men (17%) reported a medical treatment (46 of whom detailed their medication). The number of standard glasses of alcohol last week is shown in table 1. Out of the 284 men, 269 men were found to have a Tanner Stage 6, and 15 men a Tanner stage 5 of pubic hair growth, both of which are considered to be of adult quantity and type (Marshall & Tanner, 1970).

**TABLE 1:** Mean, standard deviation, median and range of investigated parameters in 284 men

Background characteristics	Mean (SD)	Median (range)
Age (years)	18.4 (0.36)	18.3 (17.5–20.5)
BMI (kg/m^2^)	23.1 (3.1)	22.6 (16.4–37.1)
Cigarette smokers (%)	21.1	
Consumption of medication last 3 months (%)	16.5	
Alcohol consumption (standard glasses per week)	5.5 (7.5)	4 (0–73)
Abstinence time (h)	60 (34)	59 (8–232)
DFI (%)	11.1 (6.1)	10.0 (3–39)
S‐Estradiol (pmol/L)	89 (24)	88 (36–180)
S‐Testosterone (nmol/L)	18 (5.2)	18 (5.9–41)
S‐FSH (IU/L)	3.4 (1.8)	3.0 (0.7–15)
S‐LH (IU/L)	4.7 (1.6)	4.4 (1.4–12)

Abbreviations: DFI, sperm DNA fragmentation index; FSH, follicle‐stimulating hormone; LH, luteinizing hormone.

### 2.4 Semen analysis

Semen samples were obtained from all subjects by masturbation into a plastic container at the laboratory. All participants were asked to keep 48–72 h of ejaculatory abstinence, and the actual abstinence time was recorded in each case as previously described (Axelsson et al., 2011; Bandel et al., 2015).

### 2.5 Sperm chromatin structure assay

SCSA is a diagnostic tool that utilizes flow cytometry to detect DNA damage in sperm samples (Evenson et al., 2002). Details of the procedure have been described previously (Bungum et al., 2004; Bungum et al., 2011; Evenson et al., 2002; Spano et al., 2000). Taken together, thawed sperm samples were normated to a concentration of 3–5 × 10^6^ sperms/ml (using a buffer containing Tris‐HCl, NaCl, and EDTA). The samples were then treated with an acid‐detergent solution (containing HCl, NaCl, and Triton X‐100) to induce DNA denaturation, after which sperm nuclei were stained with a mixture of a staining buffer (citric acid, Na_2_PO_4_, EDTA, and NaCl) and acridine orange (AO) stock solution at 1 mg/ml (equivalent amount of AO and double‐distilled water). Thereafter, the cells were analyzed with a FACSort flow cytometer (Becton Dickinson, San Jose, CA), equipped with an argon ion laser. When excited with 488 nm blue light source, AO bound to double‐stranded (intact) DNA emits green fluorescence (captured using a 515–530 nm band pass filter) whereas AO intercalated with single‐stranded (degraded) DNA emits red fluorescence (captured using a ≥630 nm filter). The ratio of red to total (red plus green) fluorescence intensity is expressed as DFI (%), that is, the extent of DNA damage in the sample, and was calculated from the DFI frequency histogram obtained from the flow cytometer (Andersen et al., 2015; Bandel et al., 2015). This was done through use of the software SCSA‐Soft (SCSA Diagnostics, Inc., Brookings, SD, USA), which initially creates a scatterplot of the DFI values of each individual sperm, after which a resulting DFI frequency profile is obtained as the histogram (Bungum et al., 2004; Bungum et al., 2011; Evenson, 2016).

Additional details on the SCSA method and outcomes were recently described (Evenson et al., 2020).

### 2.6 Statistical analysis

DFI was initially tested for normal distribution by interpretation of skewness and kurtosis, and DFI was transformed using the natural logarithm for a more normal distribution. General linear models were used to determine the association between estradiol and DFI, with estradiol as the independent variable and DFI as the dependent variable. In a second model, the subjects were divided into two groups based on estradiol levels, with the cutoff point defined as the median value of estradiol among all subjects (<88 pmol/L). The dichotomized estradiol groups were analyzed using general multivariate regression model, with the dichotomized estradiol groups as the independent variable and DFI as dependent variable. Both the analyses with continuous and dichotomized estradiol were in a second round adjusted for abstinence time (hours), cigarette smoking (yes or no), BMI, and serum levels of testosterone, FSH, and LH, similar to what appears to have been done in a previous study (Lu et al., 2018). However, we simultaneously also adjusted for alcohol consumption and medical treatment last three months (any/none). In an additional analysis, serum testosterone was replaced by free testosterone.

To evaluate a possible interaction between serum testosterone and serum estradiol, an interaction term was added into both the adjusted dichotomized and continuous models. Moreover, a 2×2 table of low/high estradiol and low/high testosterone and its effect on DFI was made to illustrate a possible interaction. Serum testosterone was dichotomized based on the median value.

Results of DFI were back‐transformed to the original scale, and therefore the mean differences along with its respective 95% confidence interval correspond to ratios. Data analyses were performed using IBM SPSS Statistics version 26‐28, with the significance level set at *p* < .05.

### 2.7 Ethical considerations

The Regional Ethical Review Board in Lund University approved the overarching study and data collection regarding factors of importance for reproductive function in men (Event No 2008/181). Participants provided a written informed consent. Each participant received 500 SEK (approximately 50 euros in 2010) as a compensation for participating in the study (Axelsson et al., 2015). Data was anonymized by replacing personal identification numbers in such a way that individual participants could not be identified from the data set and has been handled between involved researchers only.

## 3 RESULTS

### 3.1 Study population

Background data on the 284 men are shown in Table 1. Using the WHO BMI cutoff levels, 4.2% were underweight (<18.5), 72.6% were normal weight (18.5–24.9), 20.4% were overweight (25.0–29.9) and 2.8% obese (>30.0).

For the model in which the subjects were divided into dichotomized estradiol groups, the low‐estradiol group included 139 men and the high‐estradiol group included the remaining 145 subjects. The mean values of estradiol in the low‐ and high‐estradiol group were 71 and 108 pmol/L respectively.

### 3.2 Estradiol and DFI

The regression coefficient (B) from the general multivariate regression model in this analysis was back‐transformed. The regression coefficient hence represents a constant, while the independent variable (x), that is, change in estradiol, is represented by an exponent, which gives a relative change in DFI (=*B* ^*x*^). A statistically significant regression coefficient was observed in the unadjusted analysis (*p* = .02, Table 2), but the statistical significance was lost when adjustments for above‐mentioned variables were made (*p* = .08, Table 2). Consequently, DFI decreased by 0.31% and 0.28% for every pmol increase in serum estradiol in the unadjusted and adjusted model, respectively. Note that the values represent a change in percent and not percentage points. When comparing the high‐ and low‐serum estradiol groups, a statistically significant difference in DFI was observed with a 21% higher DFI in men with estradiol levels below the median value (*p* = .002) without adjustments or 18% higher DFI (*p* = .01) in the men with levels below median with adjustments (Table 3). No difference in results were observed when adjusting for free testosterone instead of serum testosterone (results not shown). Effect sizes for respective adjusted variable are represented in table 4. Besides estradiol, abstinence time also significantly affected the adjusted models (Table 4).

**TABLE 2:** Unstandardized regression coefficient (B), confidence interval (CI) and p value for association between estradiol and sperm DFI

Variables	B	95% CI	*p* value	Partial eta squared
Estradiol, unadjusted	0.9969^b^	0.9944, 0.9994^b^	.015	0.021
Estradiol, adjusted^a^	0.9972^b^	0.9941, 1.0003^b^	.079	0.011

^a^ Adjusted for abstinence time, cigarette smoking, BMI, serum testosterone, serum FSH, serum LH, consumption of medical treatment last 3 months and alcohol consumption.

^b^ Back‐transformed data.

**TABLE 3:** Differences in DFI between men with high and low estrogen in the unadjusted and adjusted dichotomized model

	Unadjusted model			Adjusted model^c^		
	Mean DFI (%)	Mean difference ratio (95% CI)	Partial eta squared	Mean DFI (%)	Mean difference ratio (95% CI)	Partial eta squared
Low estradiol (*n* = 139)	10.7	1.21 (1.07, 1.36)^a^	0.034	11.5	1.18 (1.04, 1.35)^b^	0.022
High estradiol (*n* = 145)	8.87			9.75		

Abbreviations: DFI, sperm DNA fragmentation index.

^a^ *p* = .002.

^b^ *p* = .013.

^c^ Adjusted for abstinence time, cigarette smoking, BMI, serum testosterone, serum FSH, serum LH, consumption of medical treatment last 3 months and alcohol consumption.

**TABLE 4:** *p* values and partial eta squared for respective variable in both adjusted models

	Continuous model		Dichotomized model	
Variables	*p* value	Partial eta squared	*p* value	Partial eta squared
BMI	.40	0.003	.36	0.003
Cigarette smokers	.35	0.003	.37	0.003
Alcohol consumption	.80	0.0	.93	0.0
Consumption of medication last 3 months	.54	0.001	.59	0.001
Abstinence time	<.001	0.040	.001	0.038
S‐Estradiol	.079	0.011	.013	0.022
S‐Testosterone	.97	0.0	.98	0.0
S‐FSH	.98	0.0	.88	0.0
S‐LH	.46	0.002	.50	0.002

Abbreviations: FSH, follicle‐stimulating hormone; LH, luteinizing hormone.

No statistically significant interaction between testosterone and estradiol was observed in the continuous or dichotomized models when including the interaction term (*p* = .14 and *p* = .17, respectively). When comparing the dichotomized estradiol and testosterone groups, a statistically significant difference in DFI was observed in the low‐testosterone group when comparing the high‐ and low‐estradiol groups (*p* < .001), with prominently lower DFI values in the high‐estradiol group (Table 5). No other statistically significant association was found (Tables 5 and 6).

**TABLE 5:** Impact of estradiol on DFI depending on testosterone (low/high)

	Estradiol	
Testosterone	Low	High
Low	+38% (14%, 65%), *p* < .001	Reference
High	+6.8% (−10%, 26%), *p* = .44	Reference

*Note*: The numbers indicate mean relative (%) differences (95% confidence interval of mean differences) and *p* values.

**TABLE 6:** Impact of testosterone on DFI depending on estradiol (low/high)

	Estradiol	
Testosterone	Low	High
Low	+14% (−3.6%, 36%), *p* = .12	−11% (−25%, 5.5%), *p* = .17
High	Reference	Reference

*Note*: The numbers indicate mean relative (%) differences (95% confidence interval of mean differences) and p‐values.

## 4 DISCUSSION

### 4.1 In this cross‐sectional study with 284 young men from the Swedish population, we found a statistically significant, inverse association between serum estradiol concentration and DFI

As mentioned, three previous studies have addressed the relationship between estradiol and sperm DNA integrity. Richthoff et al studied Swedish military conscripts using SCSA to assess DFI and analyzed the correlation using a bivariate model (Richthoff et al., 2002), and Meeker et al. studied the association in infertile men aged 18–54 using a multiple linear regression model, with DNA damage assessed by the neutral comet assay (Meeker et al., 2008). Both studies reported an inverse association between serum estradiol and sperm DNA fragmentation, as well as an association between testosterone and sperm DNA integrity (Meeker et al., 2008; Richthoff et al., 2002). Since serum estradiol levels may be dependent on peripheral aromatization of testosterone (MacDonald et al., 2010; Vermeulen et al., 2002), Richthoff and coworkers suggested that the observed correlation between serum estradiol and DFI may be explained by testosterone levels and not estradiol per se (Richthoff et al., 2002). In our study however, an association between serum estradiol and DFI was found in the dichotomized estradiol groups despite adjusting for testosterone. Furthermore, the association for testosterone was not statistically significant, unlike for estradiol and abstinence time, which were the only variables that were significantly significantly associated with DFI. A statistically significant association was also found between estradiol and DFI in the unadjusted multiple linear regression model, with a change of the back‐transformed regression coefficient of 0.03% in the adjusted analysis compared to the unadjusted. However, the importance of serum estradiol seemed most prominent in the low‐testosterone group. Taken together, we believe that this is the first study to demonstrate a negative association between serum estradiol and DFI in healthy late adolescents, despite adjusting for serum testosterone, with a regression coefficient seemingly robust to adjustments for additional potential confounders.

Contrary to our findings, a Chinese study on infertile men found no correlation between serum estradiol levels and DFI (Lu et al., 2018). However, that study comprised subfertile men, which may differ in comparison to men from the general population in associations between estrogens in serum and DFI as measured by the SCSA. Moreover, our study used a specific sensitive assay for measuring estradiol with a level of detection at 8 pmol/L (Axelsson et al., 2015), which may be needed in specifically men, who can have low levels (Smy & Straseski, 2018) such as below the sensitivity of the assay used in the mentioned study (Lu et al., 2018).

With the presence of ERs on all cells involved in the process of sperm production (Schulster et al., 2016), and with estrogen linked to proliferation, differentiation, and maturation of spermatids (Carreau & Hess, 2010), our results further seem to be in line with positive effects of estradiol on the male reproductive functions. Why this potential effect seemed most prominent in the low testosterone group could be due to the possibility of testosterone interacting with estradiol receptors (Rochefort & Garcia, 1976). However, because of the cross‐sectional design of this study, a causal relationship cannot be established. Further studies are therefore needed to explore the physiological role of estradiol in seminal plasma and in serum in both healthy and infertile men.

It is plausible that several limitations could have influenced the results. When analyzing estradiol and DFI with a linear regression model, the statistical significance was lost when adjustments were made. It is likely that the statistical significance could have been preserved if a higher number of men were included. Further, we used two‐tailed p‐values whereas one‐tailed p‐values have been suggested to be used when associations in a specific direction are anticipated (Ludbrook et al. 2013), like for our hypothesis. One‐tailed p‐values are half the value of two‐tailed ones, meaning that the statistical significance would have been preserved in our models if used.

Since the focus of the study was young men in Southern Sweden, the representativeness of the cohort needs to be considered. Despite a low participation rate in the cohort (14%), it is unlikely that the participants have any knowledge regarding their reproductive capability (Axelsson et al., 2011). Furthermore, no difference in levels of reproductive hormones between military conscripts who agreed to deliver a semen sample and those who declined to deliver a semen sample were reported in a previous study, indicating a similar reproductive function (Andersen et al., 2000). Selection bias towards subfertile males is therefore unlikely but cannot entirely be excluded. Thus, the cohort can be considered representative for the general population of late adolescent men in Sweden. This is a key strength of the study since it allowed us to examine the association between serum estradiol and DFI in healthy young men.

Still, a possible limitation is that only a single semen sample was delivered by each subject, because of intra‐individual variation in semen parameters. However, Rylander et al. concluded that a single semen sample is sufficient as a predictor for semen parameters including DFI (Rylander et al., 2009).

In conclusion, the result from this study supports the idea that physiologic levels of estrogens exert a protective role for sperm DNA integrity. This was however only statistically significant in men with testosterone levels below median. We hope that our results will be useful in further determination of the role of estrogens in the male reproductive system.

## AUTHOR CONTRIBUTIONS

Viktor Lu and Oscar Svensjö wrote a master thesis that was used as a first draft of this manuscript, and performed the statistical analyses. JA supervised their work and wrote the final draft. All authors agreed to the final version.
